# Recording Heart Rate Variability of Dairy Cows to the Cloud—Why Smartphones Provide Smart Solutions

**DOI:** 10.3390/s18082541

**Published:** 2018-08-03

**Authors:** Maren Wierig, Leonard P. Mandtler, Peter Rottmann, Viktor Stroh, Ute Müller, Wolfgang Büscher, Lutz Plümer

**Affiliations:** 1 Institute of Agricultural Engineering, University of Bonn, 53115 Bonn, Germany; buescher@uni-bonn.de; 2 Institute of Geodesy and Geoinformation, University of Bonn, 53115 Bonn, Germany; mandtler@igg.uni-bonn.de (L.P.M.); rottmann@igg.uni-bonn.de (P.R.); stroh@igg.uni-bonn.de (V.S.); pluemer@igg.uni-bonn.de (L.P.); 3 Institute of Animal Science, University of Bonn, 53115 Bonn, Germany; ute-mueller@uni-bonn.de; 4 Faculty of Geosciences and Environmental Engineering, Southwest Jiaotong University, Chengdu 610031, China

**Keywords:** smart device, mobile sensing, heart rate, monitoring, validation, cardiac parasympathetic, cattle, welfare

## Abstract

In the last decades, there has been an increasing interest in animal protection and welfare issues. Heart rate variability (HRV) measurement with portable heart rate monitors on cows has established itself as a suitable method for assessing physiological states. However, more forward-looking technologies, already successfully applied to evaluate HRV data, are pushing the market. This study examines the validity and usability of collecting HRV data by exchanging the Polar watch V800 as a receiving unit of the data compared to a custom smartphone application on cows. Therefore, both receivers tap one signal sent by the Polar H7 transmitter simultaneously. Furthermore, there is a lack of suitable methods for the preparation and calculation of HRV parameters, especially for livestock. A method is presented for calculating more robust time domain HRV parameters via median formation. The comparisons of the respective simultaneous recordings were conducted after artifact correction for time domain HRV parameters. High correlations (*r* = 0.82–0.98) for cows as well as for control data set in human being (*r* = 0.98–0.99) were found. The utilization of smart devices and the robust method to determine time domain HRV parameters may be suitable to generate valid HRV data on cows in field-based settings.

## 1. Introduction

The welfare and health status of dairy cows is more and more a matter of particular interest, not only for consumers, but also for dairy farmers, farm advisers and veterinarians. Consumers demand high quality and sustainable products which satisfy animal welfare and protection guidelines [1]. For livestock, welfare means the state of physical and mental harmony as well as the possibility of natural behavior in their environment [2]. Especially for high-yielding dairy cows, the reduction of stress and monitoring of their welfare status is relevant for their health and performance, as dairy cows are highly sensitive to environmental factors [3]. In addition, a reduced welfare and health status means depression in performance. At the same time, the potential of dairy cows has not been fully exploited. This in turn causes economic harm to the farmer [4]. One focus of research therefore is to recognize, solve and prevent problems in the welfare of livestock [5]. A wide range of indicators and assessment systems can be used to monitor behavior and thus evaluate well-being [6,7]. The Welfare Quality^®^ Assessment Protocol, Animal Welfare Index, German Agricultural Society (DLG)-Checklists and many more. However, to improve it, smart technologies and new instruments are needed that examine the impact of different housing conditions and environmental factors on the welfare of livestock [8]. Despite the high volume of research already published, questions about assessing welfare in cattle are still raised. This applies in particular to continuous monitoring over a longer period of time. 

Heart rate (HR) and heart rate variability (HRV) are closely associated with animal welfare [9]. Marchant-Forde et al. [10], mention the non-invasive measurement of short-term (<24 h) and long-term (≥24 h) cardiac activities that are suitable for HRV analysis, as an appropriate key provider of information for understanding the neurophysiological stress response and regulation processes in the bodies of livestock [9,11,12]. It is therefore a suitable tool for assessing the impact of current agricultural methods and systems on the welfare of animals kept in these environments. 

HR is defined as the number of beats per minute [3] and is a bio signal that continuously changes and can be detected using an electrocardiogram (ECG) [13]. The length of a heart rate cycle corresponds to the distance between succeeding heartbeats (inter-beat interval (IBI)), respectively the time measured between two proximate R peaks in ECG (RR interval) [9,14,15]. Due to permanent regulatory mechanisms, the HR and thus the IBI changes from heartbeat to heartbeat and is referred to as HRV. Factors leading to a modification of the IBI are according to Stein et al. [16], e.g., respiration, blood pressure and thermoregulation as well as daily rhythms. Measuring and analysis of HRV reflects the function of the cardiovascular autonomic nervous system [13]. The measurement parameters of the HRV are subdivided into time-based (time domain) and frequency-based (frequency domain) parameters. The analysis of HRV in time domain deals with the evaluation of IBI with regard to its variance [17]. For analysis of frequency domain, periodic variations of HR are classified to different frequencies [11,18]. This study focuses on the time domain parameters mean IBI’s (RR intervals), the standard deviation of IBI (SDNN), which shows the activity of the vegetative nervous system and the root mean square of successive differences (rMSSD) as an indicator for the parasympathetic nervous system and thus a marker for relaxation. These parameters are the most common of the variety of the existing HRV parameters and are clinical proofed [9,14,19,20]. 

The method of HRV measuring originates in human medicine and has been established over years in its ability to enable reliable statements concerning physiological states of an individual [9,11,14,19]. Furthermore, in the recent years, HRV has been used in sport medicine more and more [19,21]. Amateur athletes, for example, are using the assessment of HR and HRV to get more information about their health status and sporting progress. For this reason, there are several smart devices which entered the market quickly and made HRV analysis and the continuous collection of data on a day by day basis available for everyone [19,21]. These techniques are mostly used for short-term purposes [19]. However, especially long-term measurements are of particular importance as only with their help can information about the own well-being and internal states be made. For time series with a high variance, the selection of the time window in relation to short-term HRV measurements has a large influence on the result [11,14,22].

As documented in the literature HR and HRV in cows has been measured via portable HRV monitors (integrated in a watch) for years now [9]. Portable systems by Polar (Polar Electro Oy), originally developed and adapted to equestrian sports, are used widely [23,24], although data transmission from the watch to the sensor by means of infrared is complex, error-prone and also outdated [9,23,25]. This is due to the fact, that it is a more or less a suitable method for examinations under practical conditions as cows can easily be equipped with the sensors [23]. However, the observed animals are not used to the equipment and the chest strap for measuring moves in its position during data acquisition. This leads to more outliers compared to a measurement on humans, especially in long-term measurements [9,10,24,26,27]. Thus, the transmission accuracy is often problematic. In addition, the system has to be monitored at least two times a day for data storage and checking the correct fit of the chest strap. Therefore, the purpose of the study is to provide the usage of smart devices for HRV monitoring in cows. Smart devices for measuring HRV are already used for scientific issues. In previous studies, HRV measurements using ECGs were compared with HRV measurements using smart technologies such as photoplethysmography (PPG) or Polar HRV transmitters coupled with smartphone applications [15,19,21,25,28,29,30]. Most of the literature relates to measurements on humans, but there are also some studies that have collected data from animals (primarily dogs and cats) [31,32]. In comparison to the portable heart rate monitors by Polar, the big advantage of smart devices can be seen in the possibility to combine different services with each other. This means that further sensors can be established for the acquisition of e.g. health data on the device. Furthermore, there are advantages in the collection and storage of data and it’s possible to remotely monitor cows by live access to recorded data [29]. 

Therefore, the aim of this study was to verify if the combination of a Polar transmitter with a smartphone receiver matches the Polar portable system with regard to validity and is favorable from a practical point of view. In addition, the objective was to generate a more robust calculation of HRV parameters, especially for long-term measurements on cows with a higher rate in outliers compared to humans.

## 2. Materials and Methods

### 2.1. Experimental Probands

Data collection for this study was carried out in December 2017 at the educational and research center Frankenforst of the Faculty of Agriculture, University of Bonn, (Königswinter, Germany). The study is based on the data of two lactating, healthy Holstein-Friesian cows (Cow I and Cow II) in their first lactation with an average milk yield of (24.4 ± 1.2) kg respectively (43.1 ± 1.8) kg per day in the measurement period. They are housed on the farm in an open free-stall barn, fed a total mixed ration *ad libitium* and milked twice daily in an external milking parlor. 

During the observation time, cows were standing in a straw littered box. Further specific data on the selected focus animals are listed below. The body condition score (BCS) describes the body condition of the animals, with whose help the nutritional condition, fat deposits and indirectly the performance can be assessed:

- Cow I: BCS = 3.5; Days in Milk (DIM) = 66; not gestating
- Cow II: BCS = 3.25; DIM = 166; gestating

For checking purposes, data of a human person (♂ = 26; Height = 181 cm; Weight = 89 kg; BMI 27.2) in rest were collected likewise, on a voluntary basis. The experimental approach and handling with dairy cows in this study complies with German animal protection law. The cows are kept on an approved laboratory animal farm (39 60 03 05–547/15). 

### 2.2. Testing Procedure and Recording of Data 

**Figure 1:**
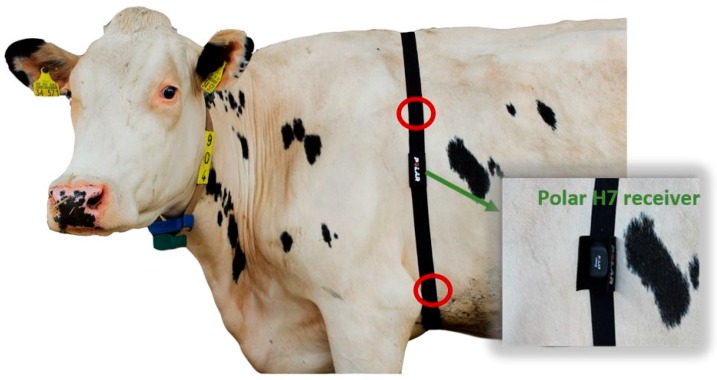
Dairy Cow I equipped with the Polar equestrian chest belt and Polar H7 receiver; red circles mark the placement of the electrodes.

For the HRV measurement, cows were equipped with an equine chest belt (Polar Equine Belt, Polar Electro Oy) cranial around the thorax. The two electrodes on the belt were placed on the left side of the body. In cattle, concerning the median plane, 5/7 of the heart are located in the left half of chest cavity and only 2/7 on the right half [33]. One electrode was located proximal to the *articulatio cubiti* and the other one accordingly on a level with the *scapula* (Figure 1). The placements of the electrodes on the skin were moistened and covered with ultrasound gel (Aquasonic 100, Parker Laboratories Inc., Fairfield, NJ, USA). The ultrasound gel warrants a secure data transmission, it establishes an optimal contact between skin and electrode and prevents embedding of air, which may have a negative effect on the transmission performance. Furthermore, conductivity can be increased through the ultrasound gel [9].

The chest strap for the human being was fixed cranial around the thorax, and the two electrodes were placed directly below the pectoral muscles located between the 4th and 5th rib. The placements of the electrodes on the skin had to be moistened with water to warrant a secure data transmission, according to the manufacturers’ specifications. 

**Figure 2:**
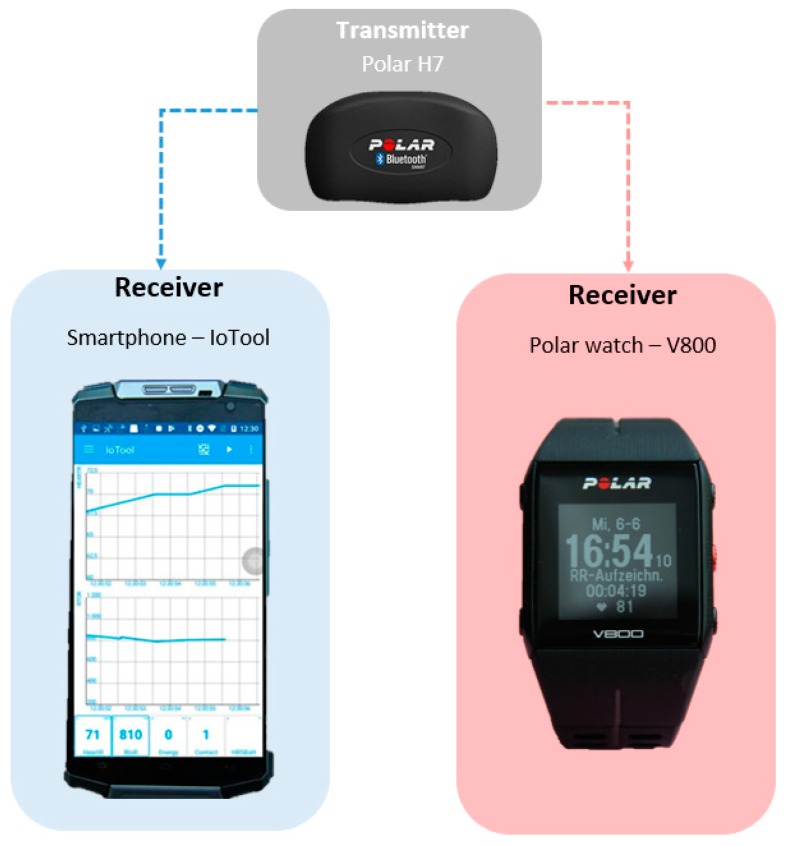
Schematic diagram of the experimental setup with Polar H7 transmitter, receiver smartphone IoTool application (**left**) and receiver Polar watch V800 (**right**).

The transmitter for measuring HRV, a Polar H7 Bluetooth capable sensor, was fixed on the belt and initially linked with the smartphone receiver, the commercially available IoTool application, (SenLab d.o.o. Ljubljana, Slovenia) via a Bluetooth connection. In a second step the transmitter could be linked with the Polar watch V800 receiver via a Polar internal connection. In this way it was ensured, that the same measured values were communicated by one transmitter sensor (Polar H7) (Figure 2). The two receivers remained in the immediate surroundings of the measured individual, according to the manufacturers’ specifications.

For each subject equipped with chest strap and Polar H7 transmitter two simultaneously measurement series were recorded, each with the smartphone IoTool application and the Polar watch V800. The data examined in this study under 3.2 refer to the recordings for Cow I over a period of 27 min for Cow II over 40 min and for human being over 60 min.

Simultaneous IBI recordings were measured for both, dairy cows and human. The Polar H7 sensor shows a sampling frequency of 1000 Hz. From these recordings the following time domain HRV parameters were derived [11,18,34]:

- RR interval: distance between two R peaks (ms);
- SDNN: standard deviation of all RR intervals; square root of variance (ms);
  (1) $$\mathrm{SDNN}=\sqrt{\frac{1}{N-1}\;\sum_{j=1}^{N}{\left({\mathrm{RR}}_{j}-\bar{\mathrm{RR}}\right)}^{2}}$$
- rMSSD: Square root of the mean of the sum of all differences between adjacent RR intervals; higher values indicate increased parasympathetic activity (ms);
  (2) $$\mathrm{rMSSD}=\sqrt{\frac{1}{N-1}\sum_{j=1}^{N-1}{\left({\mathrm{RR}}_{j+1}-{\mathrm{RR}}_{j}\right)}^{2}}$$

### 2.3. Processing of Data and HRV Analysis

The recordings of the Polar watch were transferred to a PC via Polar-specific software FlowSync 2.6.2 (Polar Electro Oy). Likewise, the data recorded by the smartphone IoTool application were stored on the same PC via USB connection. All data sets were transferred to the Matlab R2015b software (The MathWorks, Inc., Natick, MA, USA) for further processing and statistical analysis. Other statistical analysis and graphic representations were performed with the software program SPSS Statistics 25.0 (IBM, New York, NY, USA, 2017). 

As a first step outlier removal is performed following the suggestions of Marchant-Forde et al. [10] as a starting point. For this artifact correction on data from cows, successive RR intervals that differ by more than 100 ms were identified as outliers. In addition, all values outside an interval of 350 ms to 1050 ms were removed. These threshold values were defined because values outside these intervals lie in value ranges that are not physiologically possible for cows and can therefore be roughly identified as measurement errors. The HRV measures from all subjects (three data sets with *n* = 14,174 measured values) for this paper were short-term measurements and all artifacts were removed. The artifact reduction minimizes the different effects of outliers in the two different recordings.

The different devices use different communication protocols between Polar H7 transmitter and receiving units (Polar watch 1 value per second; IoTool application 1 value every 1–2 s. This results in the RR stamps drifting apart in time to a relevant extent. In order to correlate data, we start with well-founded assumption that the deviation can be described using a linear model. The offset ranged within a few seconds while the scale is a matter of hundredths of a second. The smartphone receiver with the IoTool application synchronizes to the internet time so we choose to transform the Polar watch V800 signal to align to the smartphones signal. To get good results for the transformation multiple identical data points in both recordings were manually picked. The linear equation system is then solved to get a solution for the transformation described in Equation (3). This linear model minimizes the distance between the signals depending on the number and quality of the chosen identical data points:

(3) y=mx+b

For further comparison of the recordings, data is extrapolated. Signals were interpolated to equidistant timestamps with the same frequency. We used a very high frequency with regard to Shannon’s scanning theorem and this frequency resulted from 100 times the median of the time difference between the measurements. By applying the interpolation, it’s possible to sample both signals to the same timestamps. This is important for the calculation of the correlation between the recordings. 

The Spearman-Correlation was chosen to benchmark the similarity and strength of relationship of the simultaneously recordings after checking distribution by means of the Shapiro-Wilk test. To verify the validity of the recordings, a correlation coefficient of *r* > 0.90 [35] was expected to demonstrate a strong level of linear relation of the recordings from the two receivers IoTool application and Polar watch. Using the linear regression analysis, the investigation of slope and intercept were performed additionally to show the kind of relation. The significance level was set at *p* ≤ 0.05. 

## 3. Results

### 3.1. Robust Method for Processing Time Domain HRV Parameters 

**Figure 3:**
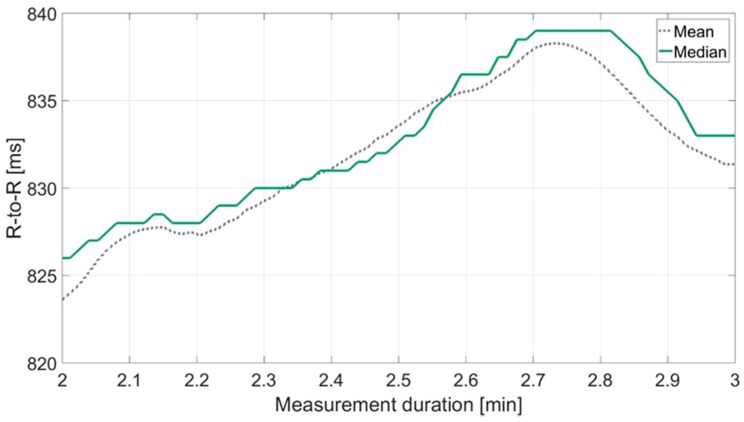
Exemplary comparison of RR interval (R-to-R) (ms) measurements prepared with mean vs. median over 60 s (*n* = 72 measured values).

Although artifacts in the recorded RR data were corrected with the method described above, outliers remained. It was observed that these still have a strong influence on HRV parameters such as SDNN and rMSSD. For this reason, a more robust estimation method for time domain HRV parameters was developed (Parameters calculated according to this method receive (*r*) as additional denotation). In contrast to rMSSD and SDNN, they are based on median and MAD rather than mean and standard deviation (Figure 3). The median is the value separating the higher half of a data sample from the lower half. The MAD is the median of the absolute deviations from the median. For $X=\left\{x_{1},\;\ldots ,x_{n}\right\}$ it is calculated by the following formula [36,37]:

(4) $$\mathrm{MAD}=median\left\{\left|x_{i}-median\left(X\right)\right|1\leq i\leq n\right\}$$

A movable filter with a length of 60 heart beats is calculated using the following formulas:

(5) $${\mathrm{MAD}}_{j}=median\left(\left|x_{i}-median\left(X_{j-29:j+30}\right)\right|j-29\leq i\leq j+30\right)$$

(6) $$\left(r\right){\mathrm{SDNN}}_{j}=1.4826*{\mathrm{MAD}}_{j}$$

(7) $$\left(r\right){\mathrm{rMSSD}}_{j}=1.4826*median\left(\left|x_{i+1}-x_{i}\right|\;j-29\leq i\leq j+30\right)$$

For the values at the beginning and end of the measurements, where the 60-beat window no longer completely covers the data, the non-robust heart parameters are extended to be able to use more data. In the case of a normal distribution, the relationship between the standard deviation SD and the MAD is estimated by the following formula:

(8) SD=1.4826∗MAD

The factor 1.4826 results from the fact that at this value the MAD is comparable to a standard normally distributed value.

This method is primarily used to determine HRV parameters in long-term measurements with a high number of outliers. The results of short-term measurements for the comparison of two HRV recording systems are presented below and this is the main focus of this study. Results for this robust method regarding long-term HRV measurements will be presented in our future publications.

### 3.2. Results for the Conformity of Measurements

**Figure 4:**
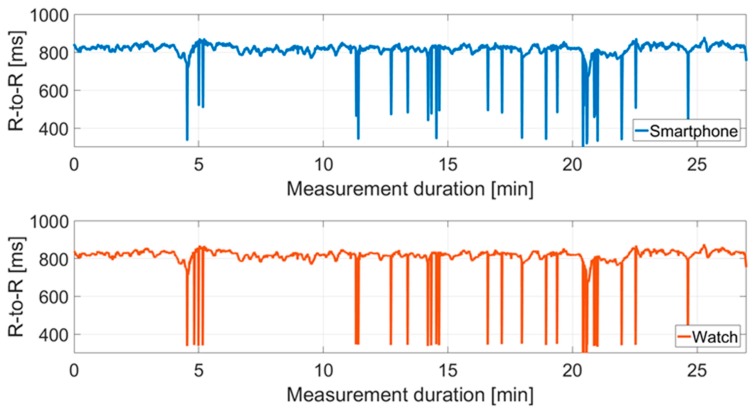
Comparison between the mean RR intervals (R-to-R) (ms) of the two signals in the raw data set derived from the smartphone IoTool application and the Polar watch V800 for Cow I over the experimental period of 27 min (*n* = 3630 measured values).

Figure 4 shows an example of a raw data set. The RR interval recordings for Cow I over the measurement period of the two different receivers are compared. 

**Figure 5:**
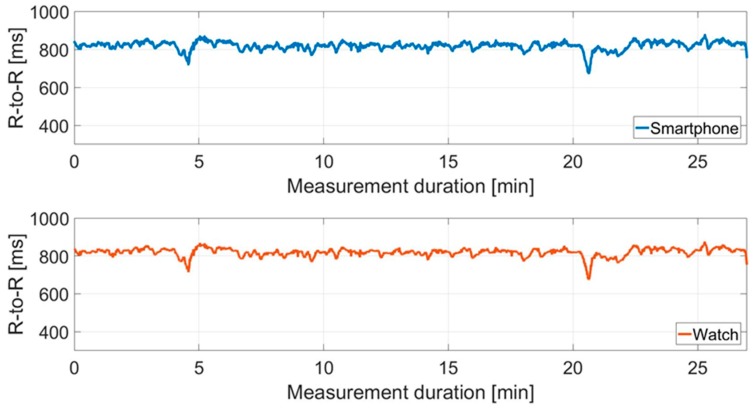
Comparison between the mean RR intervals (R-to-R) (ms) of the two signals in the data set derived from the smartphone IoTool application and the Polar watch V800 for Cow I over the experimental period of 27 min after artifact correction (*n* = 3630 measured values).

Furthermore, there are differences in the number of artifacts between and within the recordings for cows. At Cow I, 4.26% outliers were corrected for the data recorded by IoTool application. On the other hand, there is a correction of 4.93% in relation to the Polar watch data. Cow II showed a similar picture, 8.03% outliers had to be corrected for IoTool application compared to 9.94% for Polar watch data. No artifacts were recorded in either measurement (smartphone IoTool application and Polar watch V800) of the human control data set. In Figure 5 the RR interval recordings for Cow I over the measurement period are shown after artifact correction contrary to Figure 4. 

**Figure 6:**
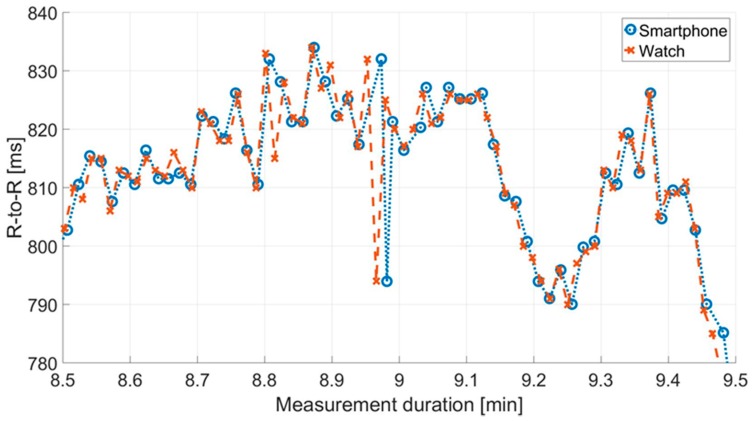
Extract of simultaneous RR interval (R-to-R) (ms) measurements for Cow I during 60 s of recording after outlier removal and but before interpolation acquired with the smartphone IoTool application and Polar watch V800.

**Figure 7:**
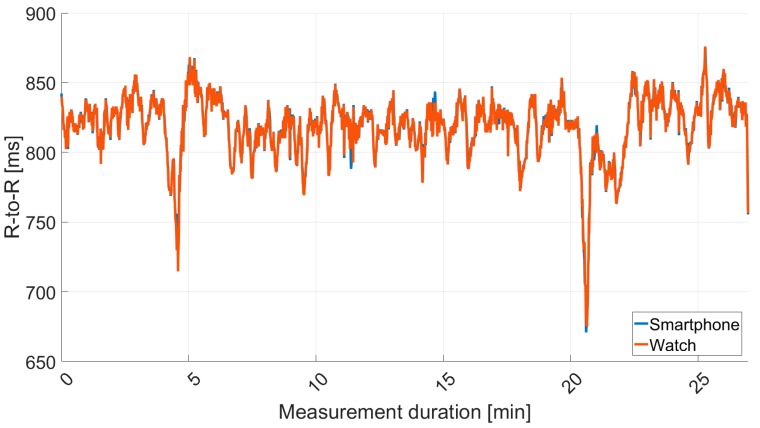
Simultaneous RR interval (R-to-R) (ms) measurements for Cow I during 27 min of recording after outlier removal and interpolation acquired with smartphone IoTool application and Polar watch V800 (*r* = 0.98; *p* < 0.001).

Figure 6 shows an extract of one data set regarding RR intervals from the smartphone IoTool application and Polar watch V800 for Cow I over 60 s before interpolating the recordings. The different timings of measurement are demonstrated. The highly interpolated superimposed RR interval recordings over measurement time of 27 min for Cow I are shown in Figure 7.

**Figure 8:**
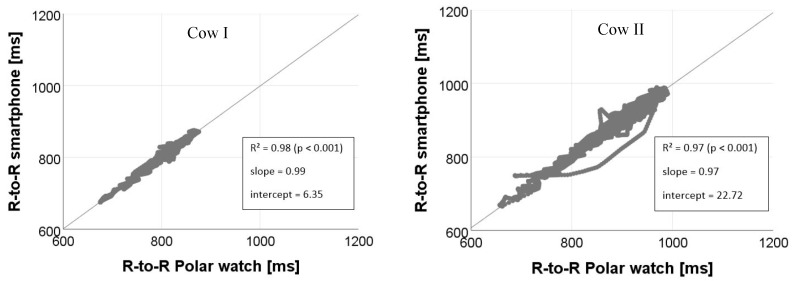
Relationships between RR intervals (R-to-R) (ms) derived from the smartphone IoTool application and Polar watch V800 for measurements of Cow I and Cow II.

**Figure 9:**
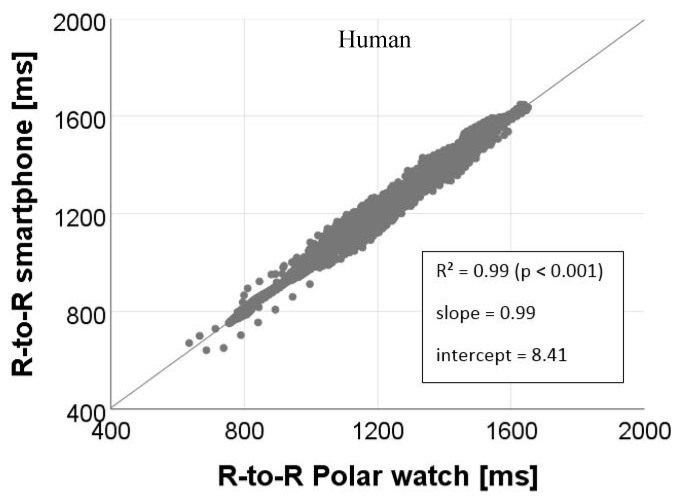
Relationships between RR intervals (R-to-R) (ms) derived from the smartphone IoTool application and Polar watch V800 for measurements on human.

The magnitudes of the correlation between smartphone IoTool application and Polar watch V800 RR interval recordings for all subjects are shown in Figure 8 and Figure 9. 

The mean values of all measured time domain HRV parameters of experimental animals (Cow I and Cow II) and the control data set collected from the human being are shown in Table 1. The correlations calculated over the respective measurement periods can also be found in Table 1 for the comparison of the two recording systems. The outcomes show that the comparability of recordings varies within HRV parameters and between individuals.

Table 2 shows the relative percentage of artifacts for time domain HRV parameters in the smartphone IoTool application to Polar V800 recordings following the suggestions of previous studies [15,29]. 

## 4. Discussion

The experiments of this study were designed to show the capability, relationship and validity of using a common smartphone application to record the most common time domain HRV parameters (RR intervals, SDNN, rMSSD) of dairy cows. The present study is the first that considers the validity of a portable HR monitor and a smartphone application linked with only one transmitter in a field-based setting. It is also the first examination of dairy cows in this way. Previous studies and concepts aimed to analyze the validity of two or more different systems measuring independent signals [15,21,22,23,24,27,28,29,30,31,32,38,39]. 

Our approach and its computed results on correlation of RR intervals, SDNN and rMSSD enables us to give a precise estimation on the similarity of the recordings received with a Polar watch V800 and a smartphone using the commercially available IoTool application. As presented in Table 1 and in regression coefficients of our studies, the validity shown on the correlation of the short-term RR interval recordings are almost perfect for cows and human being (cows: *r* = 0.98; *p* < 0.001; human: *r* = 0.99; *p* < 0.001). Our results on the agreement of the measured signals correspond to the findings of similar studies, in which a high correspondence between smartphone applications and ECG measurements was found [21,29,31,32]. For example, studies of Plews et al. [21], used a Polar H7 transmitter as well linked with a smartphone application (in-house built) in comparison to ECG measurements. They demonstrate for RR intervals collected from humans in short term measures (5 min) also clear correlations (*r* = 0.99). Cheatham et al. [29], found for two smartphone applications linked with the Polar H7 transmitter an intraclass correlation coefficient (ICC) of 0.80–0.98 for humans in short-term (5 min) resting pulse rate measurements. 

However, when looking at the HRV recordings of Cow I and Cow II, the correlations in the HRV parameter rMSSD and for Cow I also SDNN deviate from the expected results (*r* > 0.90). This is particularly interesting when it is considered that both receivers pick up the same signal sent by the Polar H7 sensor. We’re assuming, that the lower correlations in rMSSD and SDNN (*r* = 0.82–0.89) are related to the recorded artifacts in cows. In measurements of cows up to nearly 10% outliers had to be corrected whereas no artifacts have been identified in human being control data set. Jonckheer-Sheehy’s et al. [38] study shows similar results for short-term measurements (5 min) in dogs with a percentage of error up to 15.4%. When looking at Table 2, it becomes clear to what extent outliers affect the individual HRV parameters. Human percentage relative error values for SDNN and rMSSD are substantially lower (0.05%–0.49%) than for both cows (0.12%–8.67%) and can therefore also be confirmed from a comparative study by Vescio et al. [15], with a mean of 0.10% ± 0.16% relative errors of HRV measurements in short-term acquisitions. Artifact correction is crucial for HRV analysis, as artifacts have a strong influence on HRV parameters [26,39] Seely and Macklem [20] show that HRV time domain parameters SDNN and rMSSD are easy to calculate and have been clinically proven but are also sensitive to outliers. This means that artifacts must be removed before calculating these HRV parameters. This is usually done using computer-based algorithms, but often a manual review is required to eliminate all types of errors [9,19,20,40]. For example, artifacts can be caused by data collection with the help of chest straps. Other authors have rated investigations with chest straps as critical [25], especially when measuring on animals [9,10,23,24,27]. Generally, it can be said that, chest straps on cows are subject to mechanical stress. Artifacts are caused by slippage of the measuring belt respectively electrodes by e.g., movement of the animals, poor positioning of electrodes, too little ultrasonic gel as contact giver, sweating, muscle activity and even breathing movements of animals [9,23,24,25,26,32,38]. HRV recordings via ECG in animals as a reliably method also show that data is not completely free of outliers due to the above-mentioned problems of electrode slippage [23,24]. In measurements on humans similar circumstances may be responsible for the loss of contact of the electrodes and thus artifacts occurring [25,39]. However, in our data sets, we have found artifacts only in short-term HRV measurements on cows and not for humans. In the human control data set measurements for SDNN and rMSSD show perfect correlations (*r* = 1.00; *p* < 0.001). A higher artifact frequency in HRV measurements is noticeable in farm animals in particular [10,23,24,27,41]. Furthermore, sources of interference in the vicinity such as power lines and metallic objects can interfere with the transmission of data. Ille et al. and Sammito and Böckelmann [23,25] assume that for this reason radio connection from transmitter to receiver in particular is a causal problem of a high artifact frequency. We can confirm this theory with our data, based on the assumption that there were no sources of interference like the metallic feeding fences in the barn when data was collected from human being. 

Looking at Figure 4, the raw data set for Cow I shows that both receivers recorded artifacts at the same time, which is due to movements of the animal or similar. However, the artifacts of the two recording units are of different intensity. We found on average 1.3% more artifacts recorded by the Polar watch V800 than by the smartphone IoTool application for both cows. This can be attributed to the fact that the Polar watch V800 had to be linked via the weaker proprietary radio connection with the Polar H7 transmitter due to the experimental design. This means it was exposed to stronger electromagnetic interferences than the smartphone receiving unit. The Bluetooth connection of the IoTool application seems to be more robust against errors than the internal Polar connection, as Vescio et al. [15] concluded in a similar way. 

The widely used portable heart rate monitor systems by Polar have been the best option so far to collect HRV data from animals. One reason for this is that this company’s systems are widely used on the market and Polar has many years of experience in the development and production of heart rate monitor systems [25]. Previous studies have confirmed that with the Polar heart rate monitors valid HRV parameters can be determined by comparing and testing measured values by the Polar watch V800 receiver linked with Polar H7 transmitter with an ECG used as a gold standard [23,24,42,43]. In addition, the Polar H7 receiver shows the required sampling rate of 1000 Hz for recording valid HRV parameters [19,25,42]. However, the market for portable heart rate monitors of Polar is in the human sector. The systems are designed for human needs, especially for amateur athletes [19,21,25]. Broux et al. [41] come to a similar conclusion with their examination of horses. But also, Jonckheer-Sheehy [38] conclude from their outlier analysis for HRV measurements on dogs that different error types occur depending on the animal species but also possibly depending on the different measurement equipment respectively Polar model used. In this context, it should be considered that HRV data are to be evaluated and interpreted differently in cows than in humans or other animal species [24,27,37,38]. Our presented artifact model could obviously eliminate all artifacts in measurements on cows as shown in Figure 5, but we hypothesize that there are still some residual errors influencing the calculated HRV parameters (SDNN and rMSSD. Valid methods for measuring and interpreting HRV parameters in dairy cows and other farm animals are still lacking [10,12,18,27,38,39]. It must also be considered that post processing of HRV data can be a part of the remaining source of errors in the two time-series of the receiving units. This might be related to the fact that for extraction of RR intervals from Polar watch V800 the detour must be taken via the Polar FlowSync software whereas RR intervals can be transmitted directly from IoTool application. And because of the higher rate of errors more post processing is necessary for RR intervals from Polar watch V800. Lenoir et al. [39] come to a similar conclusion, they also assume that the higher post processing effort for data obtained by a Polar heart rate monitor led to poorer but acceptable correlations assessed with a Lin’s coefficient (CC Lin) in the parameters SDNN (CC Lin of 0.71) and rMSSD (CC Lin 0.84) in horses.

In sum, these above-mentioned influences probably lead to weaker but still acceptable correlations, compared to perfect correlations in the human being control data set, for the calculated HRV parameters in cows.

In addition, HRV parameters are influenced by fitness, age and gender, as well as performance and gestation stage in cows, as has already been shown in previous studies [9,18,19,44]. HRV data from cows are used in many studies as a non-invasive method to make objective statements about the health status and animal welfare [9,12,18,26]. The HRV parameter rMSSD, for example, which reflects vagal activity, can be seen as a measure of an individual’s degree of relaxation which increases with higher rMSSD values [9,11]. This plays an important role in the interpretation of the data, for example when looking at the slightly but not significantly higher mean values (Table 1) measured for this HRV parameter for all subjects. 

Long-term measurements for the evaluation of circadian rhythms in the normal housing environment of cows are crucial for answering questions about the welfare status of cows [11,14]. However, long-term measurements also increase the frequency of artifacts [15,19]. This study therefore also attempted to generate a more robust method for calculating time domain HRV parameters. The presented method for more robust algorithms for SDNN and rMSSD is especially designed for high rates of artifacts in long-term measurements. The algorithms are more robust to these as they work with the median and median absolute deviation instead of the mean which is highly afflicted by outliers [36,37]. 

The contribution of this study aims to find a suitable method to easily collect and evaluate valid HRV data from cows. The combination of the valid Polar H7 sensor with a smartphone application brings significant advantages and shows good correlations to standard methods in HRV measurements, as we have shown, as well as other studies [21,29,31,32]. The main purpose of this study is to present smart solutions with the aid of collecting data concerning the animal welfare situation in field-based settings, especially for long-term measurements. The advantages of the presented smart device for the collection of HRV data are as follows. The data recorded via smartphone IoTool application can be controlled live from outside without having to remove the receiving unit from the cow in comparison to the Polar watch receiver. Furthermore, it is possible to receive for example error messages via the network in which the recording smart device can be integrated. Thus, the risk of data loss due to transmission interference, bad contact of the electrodes and system failure can be reduced to a minimum. For clarifying questions regarding the animal welfare situation, the HRV online monitoring of cows brings benefits. Animals can move and interact undisturbed by human influences in their environment [45]. Another advantage of using smart technologies can be seen in their rapid further development. The systems are becoming more and more precise, the market is large and flooded with user-friendly applications for HRV measurements [21,25,29]. Additional sensors can be easily linked and installed on smart platforms and only one device is needed to gather behavior and health data of animals [21], instead of several individual sensors on livestock nowadays like pedometers, sensors for animal identification and so forth [46]. These additional sensors implemented on a smartphone could be, for example, an accelerometer for the detection of lying behavior or tracking systems for determining a cows’ position. Data can be easily stored and integrated via network communication in the herd management system [46]. In this way, objective access to a variety of health data on farm animals can be provided, this can help with the welfare status and the comfort situation of livestock can be better addressed in the future. In turn, it means that smartphones respectively smart devices offer smart solutions and interfaces to capture the complex effects that affect the welfare status of dairy cows.

Generally, it can be said, that smart devices can simplify acquisition of HRV in livestock in field-based settings because they are essentially user-friendly and e.g., HRV data can be checked directly on the device without using further software programs [21]. In addition to clarifying scientific questions, other users, such as veterinarians, can also carry out HRV measurements on cows in a simple manner [29,32].

In future studies it’s necessary to identify the different types of errors that occur in HRV measurements on cows via ECG measurements. Marchant-Forde et al. [10] and Jonckheer-Sheehy et al. [38] identified different types of errors for pigs and dogs, assuming that these are largely but not completely identical between animal species. Developing methods for dealing with outlier removal in HRV time series are essential as well as methods for the interpretation of HRV for cows, especially regarding long-term measurements.

Furthermore, the artifact rates for HRV measurements on cows should be improved in general. For this purpose, implants may be tested to collect HRV data, as they are less affected by outliers than chest strap systems [9]. 

## 5. Conclusions

In this study, we present a valid and simple method for HRV recordings in cows. We compared a portable Polar heart rate monitor as a standard method of measuring inter-beat intervals of dairy cows with a customized smartphone application. Both receivers could simultaneously record the same measured signal. To our knowledge, this is the first time this technology has been tested on cows. Correlations *r* > 0.90 for cows and human being as well as a linear relationship with a slope near to 1.00 were found between the Polar system and the smartphone application. The usage of a smartphone application as receiver allows for online monitoring and less needed contact on the animal. Moreover, the usage of a smart device allows to connect multiple sensors to one device. The presented IoTool application therefore, offers many interfaces for different sensors. This helps for digitization in the environment of dairy cows and will therefore be able to provide smart solutions for answering questions about the welfare of dairy cows in the future. Different research questions can be thought of where a smart device as recording unit for different sensors will be helpful. This study also attempted to provide a new method for valid processing of more robust time domain HRV parameters for cows in long-term measurements to evaluate the animal welfare. 

## Figures and Tables

**Table 1:** Summary statistics of heart rate variability (HRV) parameters (mean ± SD) and validity statistics (correlation) measured via Polar watch V800 and smartphone IoTool application.

	Smartphone IoTool Mean ± SD	Polar V800 Mean ± SD	Spearman Correlation (*p* Value)
Dairy Cow I			
RR interval (ms)	819.08 ± 21.96	818.08 ± 22.91	0.98 * (<0.001)
SDNN (ms)	15.36 ± 8.31	14.67 ± 8.54	0.89 ^†^ (<0.001)
rMSSD (ms)	7.82 ± 1.86	7.41 ± 1.77	0.82 ^†^ (<0.001)
Dairy Cow II			
RR interval (ms)	899.84 ± 42.03	897.41 ± 44.91	0.98 * (<0.001)
SDNN (ms)	26.86 ± 15.15	26.33 ± 15.30	0.97 * (<0.001)
rMSSD (ms)	10.15 ± 2.22	9.34 ± 1.96	0.88 ^†^ (<0.001)
Human control data			
RR interval (ms)	1326.13 ± 123.08	1324.68 ± 125.86	0.99 * (<0.001)
SDNN (ms)	88.07 ± 43.73	88.50 ± 44.48	1.00 * (<0.001)
rMSSD (ms)	73.63 ± 12.73	73.59 ± 12.63	1.00 * (<0.001)

RR interval: distance between successive R waves; SDNN: standard deviation of all RR intervals; rMSSD: Square root of the mean of the sum of all differences between adjacent RR intervals; * good validity; ^†^ moderate validity.

**Table 2:** Percentage relative errors of heart rate variability (HRV) parameter measurements.

Error %	Smartphone IoTool vs. Polar V800
Dairy Cow I	
RR interval (ms)	0.12
SDNN (ms)	4.70
rMSSD (ms)	5.53
Dairy Cow II	
RR interval (ms)	0.27
SDNN (ms)	2.01
rMSSD (ms)	8.67
Human control data	
RR interval (ms)	0.11
SDNN (ms)	0.49
rMSSD (ms)	0.05

RR interval: distance between successive R waves; SDNN: standard deviation of all RR intervals; rMSSD: Square root of the mean of the sum of all differences between adjacent RR intervals.
